# Evaluation of Bioactive Glass Treatment for Dentin Hypersensitivity: A Systematic Review

**DOI:** 10.3390/biomedicines11071992

**Published:** 2023-07-14

**Authors:** Dorotea Petrović, Dora Galić, Davor Seifert, Nikolina Lešić, Martina Smolić

**Affiliations:** 1 Department of Dental Medicine, Faculty of Dental Medicine and Health, J.J. Strossmayer University of Osijek, 31 000 Osijek, Croatia; 2 Faculty of Dental Medicine and Health, J.J. Strossmayer University of Osijek, 31 000 Osijek, Croatia; 3 Department of Translational Medicine, Faculty of Dental Medicine and Health, J.J. Strossmayer University of Osijek, 31 000 Osijek, Croatia; 4 Department of Pharmacology, Faculty of Medicine, J.J. Strossmayer University of Osijek, 31 000 Osijek, Croatia

**Keywords:** bioactive glass, calcium sodium phosphosilicate, dentin hypersensitivity, fluoro calcium phosphosilicate

## Abstract

The aim of this systematic review is to compare home and office desensitizers containing bioactive glass with control groups in randomized controlled trials (RCT) conducted between 2018 and 2022. According to PRISMA guidelines, three electronic databases (Scopus, PubMed, and Cochrane Library) were searched for published scientific articles in October 2022. RCT with adult participants with dentin hypersensitivity (DH) diagnosed by evaporative, mechanical, or thermal stimulation, with a follow-up period and quantified pain assessment were included in the study. Studies that reported DH due to tooth restoration, crown preparation, bleaching, or periodontal surgery or used bioactive glass-ceramics were excluded. The quality of the studies was assessed using version 2 of the Cochrane Risk-of-Bias Tool for randomized studies (RoB 2 tool). Articles that were duplicative or unrelated to this study were excluded. Nine articles were selected for full-text evaluation, whereas two articles were rejected. The remaining seven reports were included in this review. The calcium sodium phosphosilicate group (CSPS) was not significantly different from the positive control groups. Compared with the control groups, fluoro calcium phosphosilicate (FCPS) may be the most effective long-term treatment option. In terms of DH symptom reduction, the FCPS group performed better than the CSPS group. CSPS at a concentration of 5–15% and FCPS at a concentration of 5% are effective in treating DH in adult participants.

## 1. Introduction

Bioactive glass (BAG) is a biomaterial commonly used in dentistry due to its biocompatibility, bioactivity, and antimicrobial properties [1,2]. The original bioactive glass, later known as Bioglass 45S5^®^, was developed by Larry Hench at the University of Florida in 1969 [3]. Hench’s original goal was to create a bone regenerating material, as polymer and metal implants, which were intended to be chemically inert, were rejected at the time due to fibrous encapsulation that hindered integration into the recipient’s tissue [1]. SiO_2_, Na_2_O, CaO and P_2_O_5_ make up 46.1 mol% of Bioglass 45S5^®^, 24.4 mol% sodium oxide, 2.6 mol% calcium oxide and 2.6 mol% phosphorus pentoxide [4]. After 40 years of research into bioactive glasses, no other bioactive glass composition has been discovered that offers better biological capabilities than the original composition of Bioglass 45S5^®^ [3]. Since its approval by the Food and Drug Administration (FDA) in 1985, it is estimated that Bioglass^®^ 45S5 has been used to repair bone and tooth defects in more than 1.5 million patients [5]. In dentistry, it is widely used as a material for bone grafts and implants, as well as for enamel remineralization and, most recently, for the treatment of dentin hypersensitivity. BAG is also used for restorative materials, air abrasion, direct pulp capping, and root canal treatment [2].

Dentin hypersensitivity (DH) is a common condition, with a prevalence of 10 to 30% in the general population, making it one of the main issues in dental practice. The impression of pain in oral disorders, including DH, is significant in comparison to the actual source of the pain when compared to other sections of the body. According to sufferers, DH-related discomfort is so troublesome that it makes it difficult to eat, sleep, or even work. The agony manifests suddenly, yet is sustained for a long period of time by a sizable proportion of patients. Gibson et al. propose that DH should be regarded as a chronic condition due to the persistence and repetition of pain over such protracted periods of time. The impact on social and family life, as well as on the ability to work and make a living, are all clear indicators of consequences. Thus, conducting meaningful assessments of chronic pain is a difficult undertaking, both in clinical practice and in research on chronic pain management [6].

DH mostly affects premolars and incisors, and is defined as a brief and transient severe pain triggered by a thermal, osmotic, chemical, or mechanical stimulus that cannot be associated with any other dental pathology. Therefore, a diagnosis of exclusion is conducted. The differential diagnosis must take into account several clinical conditions, such as postoperative or broken restorations, cracked tooth syndrome, bleaching sensitivity, caries, and pulpitis, which can mimic DH symptoms [7]. A clinical examination (radiographic examination, percussion test, and vitality test) and an evaluation of the patient’s response to a stimulus are also used to detect dentin hypersensitivity. These techniques for evaluating the patient’s response include the use of a probe for tactile or mechanical stimuli, cold water at different temperatures, a heat test (with hot water or blowing air), the use of a sucrose solution for an osmotic test, and devices such as the Yeaple probe and the scratch device [8]. Verbal rating scales (VRS) that assign numerical scores to various pain descriptors are used to assess pain intensity in DH measurements. Scores are often assigned randomly, which calls into question the mathematical interpretation of the rating system. To address the shortcomings of the VRS, the visual analogue scale (VAS) was developed. The patient must indicate his or her pain level along a 10-cm line using descriptors representing the absolute minimum and absolute maximum pain intensity [9]. The predisposing factor for DH is exposed dentinal tubules in conjunction with gingival recession and loss of tooth structure such as cementum or enamel [7].

The peak age for a diagnosis of DH is thought to be between the third and fourth decades of life, and women appear to be more commonly affected than men. It is not known what role age plays in the distribution or frequency of DH. In contrast to DH in older patients, which is more commonly caused by exposed root surfaces in periodontal disease, erosions with exposed dentin appear to be more common in younger adults [10]. This disorder severely limits the patient’s ability to speak, eat, drink, and brush their teeth. It also adversely affects oral health-related quality of life [11]. In addition, more severe DH lasting longer than six months can cause psychological and emotional disturbances that may lead to the development of chronic dental pain [12].

Several theories have been proposed regarding the mechanism of pain in DH. According to the odontoblastic transduction theory proposed by Rapp et al., odontoblasts serve as receptor cells that send a membrane potential to the nerve endings of the pulp at the pulpodentine border, where it causes pain. This claim is invalid because there is no evidence that synapses exist between odontoblasts and nerve terminals. The direct innervation theory, by contrast, states that sensory nerve endings extend from the pulp to the dentin-enamel junction. An action potential is triggered by direct mechanical stimulation of this nerve terminal. However, there is no evidence that neural cells exist in the superficial dentin [8].

Brannstrom and Astrom proposed the hydrodynamic theory to explain the pain that occurs in DH. According to the hydrodynamic theory, various external stimuli cause fluid movement within the exposed dentinal tubules, resulting in the activation of nerve endings and pain [13].

However, due to the variety of treatment methods available, there is no gold standard for the treatment of DH. Since DH is caused by exposed dentinal tubules, closure of these tubules is the basis of any DH treatment. There are several treatment approaches, including non-invasive physical and chemical occlusion of the exposed dentinal tubules, nerve desensitization, and photobiomodulation [14,15]. 

The main mechanism of nerve desensitization is based on intradental depolarization of nerve terminals using potassium salts, most commonly potassium nitrate, to prevent the transmission of action potentials. As a result, there is an improvement in symptoms as the brain perceives the pain as less intense. On the other hand, there are two types of photobiomodulation treatments based on the intensity of laser power used: Low intensity lasers (e.g., gallium aluminum arsenide laser (GaAlAs) or helium–neon laser (HeNe)) and high intensity lasers (e.g., neodymium-doped yttrium aluminum garnet laser (Nd:YAG), erbium-doped yttrium aluminium garnet laser (Er: YAG), erbium, chromium: yttrium, scandium, gallium, garnet laser (Er, Cr: YSGG) and CO_2_). High intensity lasers are used to obliterate the dentinal tubules by inducing the formation of secondary and tertiary dentin via odontoblasts, whereas low intensity lasers disrupt the Na^+^/Ca^2+^ exchanger in the cell membrane, preventing the transmission of pain stimulus [14,16]. Fluid movement and the resulting activation of nerve fibers can both be reduced via physical constriction of the dentinal tubule [17]. Therefore, occlusion of the dentinal tubules, which requires the incorporation of particles into the dentinal tubules, is a third treatment option for dentin hypersensitivity. Fluorides, oxalates, and arginine are examples of chemical agents used to treat DH in addition to mechanical agents such as adhesives, hydroxyapatite, and bioactive glass [14,16].

BAG which binds to collagen fibers and deposits a layer of hydroxyapatite (HAP) to further seal the dentinal tubules and resist the effects of the acidic environment, helps prevent physical occlusion of the tubules. More specifically, the initial reactivity leads to the formation of a negative surface charge on the particle surface, which allows binding to the side groups of exposed type I collagen fibers, which are numerous in the exposed dentin [18]. The mechanism of HAP formation consists of several steps. It begins with the exchange of Sodium and calcium ions on a glass surface with hydrogen cations from the surrounding body fluid. The increase in OH concentration, and thus pH, causes the silicate network of glass to dissolve and silanol groups to form in the surrounding fluid, which condenses and forms a polymerized silica gel on the glass surface. The silica gel provides a large number of precipitation sites for heterogeneous nucleation of calcium and phosphate ions and forms an amorphous calcium phosphate layer. Finally, it absorbs carbonate ions from body fluid, leading to crystallization of HAP [2]. An in vitro study using scanning electron microscopy confirmed previous hypotheses that BAG and fluoride-containing BAG successfully close the dentinal tubules by forming a HAP layer [19]. Based on their meta-analysis, Martins et al. claimed that BAG, or more precisely calcium sodium phosphosilicate (CSPS) seems to be the most effective method to form apatite minerals and reveal DH symptoms compared to all other existing agents for the treatment of DH [20]. 

Since 2004, Bioglass 45S5^®^ particles have been used in toothpaste under the name NovaMin^®^ [5]. The FDA-approved fluoride-free daily toothpaste containing 5% NovaMin^®^ (Oravive^®^) was the company’s first Bioglass product. It was developed to rapidly and continuously reduce the sensitivity of dentin [21]. In the year 2010, new products became available in the market, such as Sensodyne^®^ Repair and Protect formulations, which are sold in over 20 countries. Due to the recent success of NovaMin^®^ toothpaste, novel glass compositions have been developed, including fluoride-containing bioactive glasses that can release fluoride ions and promote the formation of more acid-resistant fluorapatite instead of HAP on dentin. BioMin F^®^, a fluoride-releasing bioactive glass, has a higher phosphate content, CaF_2_ in the glass and a smaller average particle size compared to NovaMin^®^. BioMin F^®^ toothpaste was launched in the UK, Germany and India in 2016 [5].

Several authors have conducted systematic reviews in this research area. The qualitative synthesis by de Freitas et al. [22] included not only randomized controlled trials (RCT) using CSPS as the experimental group but also Biosilicate^®^, a bioactive glass-ceramic. In addition, Zhu et al. [23] performed a meta-analysis on the effect of using only CSPS on DH. Since recent research has also used fluoro calcium phosphosilicate (FCPS) as an experimental group for DH treatment and bioactive glass and bioactive glass-ceramics do not have the same properties [24], we have decided to include in this review all relevant literature on the subject of bioactive glass (CSPS and FCPS) and to exclude bioactive glass-ceramics as an experimental group.

In order to synthesize newly obtained data in this research area, we include all relevant literature published within the last five years in this review. The objective of this review is to evaluate current research on the use of at-home and in-office desensitizing agents containing BAG (CSPS and FCPS) compared to negative and positive control groups in adult participants. The null hypothesis is that BAG does not reduce the symptoms of DH compared to the control group.

## 2. Materials and Methods

This systematic review followed the guidelines of the Statement for Reporting Systematic Reviews and Meta-Analyses of Studies (PRISMA) [25]. The review was registered at INPLASY.

### 2.1. Eligibility Criteria

Randomized clinical trials of any duration published between 2018 and 2022 involving adult participants older than 18 years diagnosed with DH from evaporative, mechanical, or thermal stimulation were included in this systematic review. Included studies had an experimental group containing bioactive glass and a control group with a placebo or desensitizing agent that did not contain bioactive glass. Furthermore, patient follow-up and quantified pain ratings were required for inclusion in this report. Studies that reported DH due to tooth restoration, crown preparation, bleaching, or periodontal surgery were excluded. This study also excluded clinical trials that used bioactive glass-ceramics. PICO criteria are shown in Table 1.

### 2.2. Search Strategy

For this systematic review, three electronic databases (Scopus, PubMed, and Cochrane Library) were manually searched for published scientific articles on 10 October 2022, with a limit from 2018 to 2022 regarding the age of publication. The authors searched for terms: “bioactive glass” or “phosphosilicate” along with the term “dentin sensitivity”. There was no language limitations. The gray literature was not searched. 

### 2.3. Selection Process

The titles and abstracts of the collected articles were initially screened by three reviewers. For studies that appeared to meet the inclusion criteria, the full texts were collected and analyzed independently. Finally, the inclusion and exclusion criteria were used to determine whether a study was eligible. Any disagreements between reviewers were discussed during the study selection process.

### 2.4. Data Extraction

Full-text data were extracted from the selected eligible articles. After double-checking for accuracy, the extracted data were compared. We collected data on the report (author, publication year, title), participants (number, age), and intervention (sensitivity measures for eligibility criteria, home/office application, application instructions, pain assessment scales with type of stimulation, experimental and control groups, duration of follow-up, and outcomes).

### 2.5. Study Risk of Bias Assessment

The quality of the studies was assessed according to the Revised Cochrane risk-of-bias tool for randomized trials (RoB 2) [26] using the following parameters: (1) randomization process bias; (2) deviation bias from planned interventions; (3) missing outcome data bias; (4) outcome measurement bias; and (5) reported outcome selection bias. The authors used the instrument independently for each included study and recorded supporting information to assess the risk of bias in each domain (low risk; some concern; high risk). Any disagreements in the assessment of risk of bias were resolved through discussion to reach consensus. Using the RoB 2.0 guidelines [26], an overall assessment of risk of bias (low risk; some concern; high risk) was made for each specific outcome.

## 3. Results

### 3.1. Study Selection

**Figure 1:**
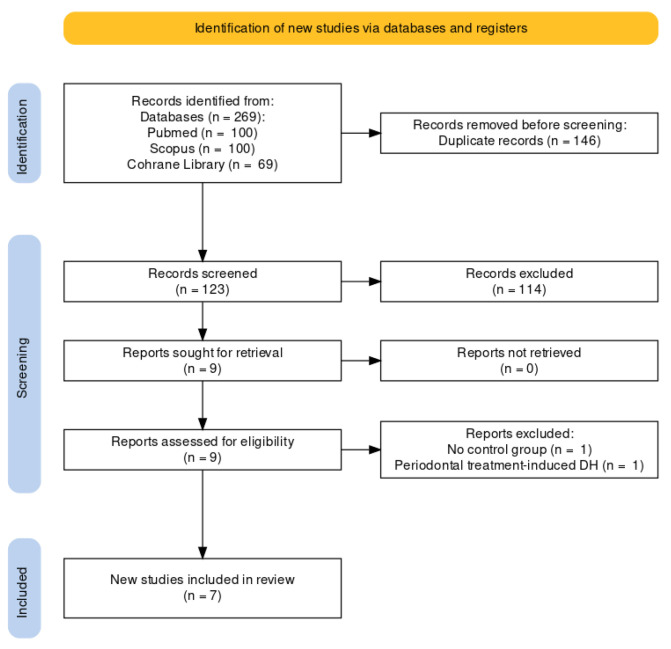
The Prisma Flow Diagram.

The initial search of all sources yielded 269 entries. Before screening, duplicated articles (146 entries) were removed. After screening titles and abstracts, articles unrelated to this systematic review (114 entries) were eliminated. As a result, nine articles were retained for the full-text review, whereas two articles were excluded (no control group, DH caused by periodontal treatment). The remaining seven reports were included in this review [27,28,29,30,31,32,33]. A flowchart of the study selection process is shown in Figure 1.

### 3.2. Studies Outcomes

A small number of studies were included in this qualitative synthesis (n = 7), four of which used CSPS for the experimental group [27,30,31,32], two used FCPS as the experimental group [28,33], and one study compared the effects of these two products [29]. A summary of the studies included in the systematic review is shown in Table 2.

CSPS was not significantly different from certain positive control groups (15% nano-HAP, 10% nano-HAPKN (nano-HAP supplemented with potassium nitrate), Nd:YAG laser, fluorinol toothpaste), so they may be complementary in terms of alleviating DH pain [27,30,32]. Compared to 10% nano-HAP, CSPS reduced DH significantly more at six and eight weeks [27]. However, fluorinol toothpaste performed better at three and four weeks to tactile stimulation. Namely, it reduces dentin permeability by precipitating calcium fluoride in the dentinal tubules [30].

*Pro-argin^®^* and strontium acetate are efficacious in relieving DH pain in the short term, but FCPS may be the best long-term treatment option [28], as shown by Patel et al. [33] after 1 month when visual analogue scale (VAS) scores in the FCPS group were found to be significantly better when compared to the *ProArgin^®^* and placebo toothpastes in the treatment of DH.

According to Ashwini et al. [29], the FCPS group was more effective than the CSPS and standard fluoride dentifrices in reducing DH symptoms.

### 3.3. Risk of Bias

We used the RoB 2.0 tool [26] to assess the risk of bias in all of the included studies. Table 3 provides a summary of these assessments for each of the five individual domains of the Risk of bias assessment. There were some concerns about the overall risk of bias, with three of the articles rated as having a high risk of bias and two rated as having some concerns.

## 4. Discussion

Clinical applications of BAG in dentistry include applications in implantology, oral surgery, periodontology, bone regeneration, pulp capping, and root canal treatment. It can be used as a restorative material, as a dental adhesive, for remineralization of tooth enamel, and for tooth hypersensitivity [2].

BAGs are promising additions to restorative dentistry because they have the ability to raise local pH, release beneficial ions (such as Ca^2+^, PO_4_^3−^ and F^−^), and promote the formation of apatite [34].

According to Splieth and Tachou [10], dentin hypersensitivity is a clinically relevant and widespread issue affecting a quarter of the adult population. Their overall quality of life may be significantly affected by limitations in daily activities. On the other hand, relatively little research has been published on DH [35]. Dentistry thus faces the problem of reducing dentin hypersensitivity. Therefore, it is highly desirable to produce alternative products that can obliterate the dentinal tubules and are resistant to chemical and mechanical stress [36]. The materials of the future are bioactive glasses. The effectiveness and usability of this material are limited only by the creativity and originality of researchers. Developing more affordable coating techniques is a critical step in making the bio-devices researchers obtain available to as many people as possible [37].

In this systematic review, desensitizing agents containing BAG were shown to be adequate for the treatment of DH. However, the included randomized controlled trials lack standardization and study data.

As inclusion criteria, investigators used either VAS or a Schiff scale score limited to different pain values, as shown in Table 2. CSPS concentrations ranged from 5 to 15% [27,29,30,32], while FCPS concentrations were consistently 5%. Two studies [28,31] using *NovaMin^®^* and *BioMinF^®^* did not provide CSPS and FCPS concentration data. CSPS and FCPS can be used as at-home and in-office interventions in the form of dentifrice or prophylaxis paste. All included studies used over-the-counter toothpaste, except for one study [32] that was an in-office intervention. Two studies used a modified Bass brushing technique [28,33], and one used the Stillman method [30], while the others did not report any specific technique. Furthermore, some investigators provided participants with soft-bristled toothbrushes [27,29,30,33].

The study duration for the observed articles ranged between four and eight weeks, while the number of participants ranged from 20 to 140. Some randomized clinical trials included a one- to two-week washout period to allow each participant to begin on the same toothpaste background [27,30]. Furthermore, some of them included root planning and/or scaling as pretreatment [29,31,33].

All studies used a negative control, positive control, or a combination thereof. Arshad et al. [28] determined that the use of fluoride toothpastes as a negative control is one of their study’s limitations, because these toothpastes might have therapeutic effects on DH. Aside from this study, another included study used a negative placebo dentifrice containing fluoride in their composition [29], while one did not specify which placebo paste was used [33]. Toothpastes used in the washout period also contained fluoride [27,30].

In the clinical trials, the pain was assessed using the VAS and the Schiff scale. The VAS was used after evaporative [27,30,32,33], thermal [27,28,29,30], and tactile stimulation [28,30,32] or to assess subjective sensitivity [29], whereas the Schiff scale was only used in one study to assess pain after thermal stimulation [28]. There are different methods for stimulating DH with each of the above stimuli that should be taken into account. According to Maximiano et al. [32], evaporative stimulation is more precise than tactile stimulation because the air reaches the exposed dentin at the same time, whereas tactile stimulation uses a probe that must touch a specific area of exposed dentin to elicit pain, which often clinically differentiates. Two studies [30,33], in addition to pain measurement, also measured the gingival and plaque index.

Some studies imposed restrictions such as a time limit on eating after brushing teeth, teeth whitening [27], taking acidic foods and drinks before the measurement [28], and the use of other oral hygiene products [27,28,29]. Only two studies [27,29] emphasized that there were no adverse effects of the paste, whereas the others did not provide information on whether there were any potentially negative effects.

We included all studies in this review, regardless of the risk of bias. Amaechi et al. had a high risk of bias due to the per-protocol analysis, whereas the other included studies were either intention-to-treat or modified intention-to-treat analyses [27]. Bala et al. [31] were also at high risk because the study was single-blinded. All other included studies were double [27,29,30,32] or triple [28,33] blinded. Finally, Patel et al. [33] demonstrated a high risk of bias because this randomized controlled trial used non-random sampling.

This study has certain limitations such as a small number of included studies (n = 7), a high risk of bias (n = 3), and variability and heterogeneity in clinical research methodology. For future studies, we recommend the standardization of DH detection procedures, both for the comparison of data in future studies in this research area, and especially for the systematization of DH detection in general. To evaluate the clinical therapeutic effect of BAG on DH and its effects on adhesion repair more objectively and truthfully, we believe it is necessary to conduct further clinical studies in the future.

## 5. Conclusions

One of the main reasons for dental treatment is the pain caused by dentin hypersensitivity, which can affect a person’s quality of life. Within the study’s limitations, it was determined that CSPS in concentrations of 5–15% and FCPS in concentrations of 5% through at-home or in-office dental applications are effective for managing dentin hypersensitivity in adult participants.

## Figures and Tables

**Table 1:** PICO criteria.

**Patient and Population (P)**	Human and Animal studies
**Intervention (I)**	Application of bioactive glass materials
**Comparator or control group ©**	Application of placebo or desensitizing agent that did not contain bioactive glass
**Outcomes (O)**	Reduction of DH

**Table 2:** A summary of included studies.

Author & Year	Title	Number of Participants	Ages of Participants	Sensitivity Measurements	At-Home/ In- Office Application	Application Instructions	Pain Assessment Scales & Type of Stimulation	Experimental Group	Control Group	Follow-Up Period	Results
**Amaechi et al., 2021** [27]	Clinical efficacy of nanohydroxyapatite-containing toothpaste at relieving dentin hypersensitivity: an 8 week randomized control trial	105	18–80	Evaporative stimulation followed by VAS scores ≥2 on at least one tooth	At home	Brushing 2× daily for 2 min with a soft-bristled toothbrush and rinsing with 10 mL of water for 10 s.	VAS–evaporative and thermal stimulation	15% CSPS toothpaste (Sensodyne™ Repair & Protect with NovaMin^®^)	10% nano-HAP toothpaste/15% nano-HAP toothpaste/10% nano-HAP toothpaste with 5% potassium nitrate	Baseline, two, four, six, and eight weeks	CSPS was not significantly different from 15% nano-HAP and 10% nano-HAPKN. When compared to 10% nano-HAP, CSPS reduced DH significantly more at 6 and 8 weeks.
**Arshad et al., 2021** [28]	Comparative efficacy of BioMin-F, Colgate Sensitive Pro-relief and Sensodyne Rapid Action in relieving dentin hypersensitivity: a randomized controlled trial	140	18–50	Evaporative stimulation followed by Schiff scores ≥2 for at least two tooth	At home	1 min topical application with the finger about half an inch length on dry tooth surface followed by brushing with a modified bass method.	VAS-tactile, and thermal stimulation (20/35)/Schiff–thermal stimulation (32/35)	FCPS dentifrice (BioMin F^®^)	8% Pro-Argin™ dentifrice (Colgate^®^ Sensitive Pro-Relief™)/8% strontium acetate dentifrice (Sensodyne Rapid Action™)/Placebo sodium fluoride dentifrice (Colgate^®^ Total)	Baseline, immediately, three days, two, four, and six weeks	FCPS dentifrice is an effective long-term treatment option for DH. Dentifrices containing Pro-Argin^TM^, and strontium acetate are effective for immediate relief from DH pain.
**Ashwini et al., 2018** [29]	Comparative evaluation of desensitizing efficacy of dentifrice containing 5% fluoro calcium phosphosilicate versus 5% calcium sodium phosphosilicate: A randomized controlled clinical trial	60	18–35	Subjective sensitivity and thermal stimulation followed by VAS scores ≥4 on at least two tooth	At home	Brushing 2× daily for 2 min with soft bristled toothbrush and with an amount equal to about half the length of the bristle head.	VAS–typical subjective sensitivity and thermal stimulation	5% FCPS dentifrice/5% CSPS dentifrice	Fluoride dentifrice	Baseline, immediately after scaling and root planning, 15, 30, and 60 days	The FCPS group was more effective in reducing DH, followed by CSPS.
**Bhowmik et al., 2021** [30]	Comparative evaluation of fluorinol and calcium sodium phosphosilicate-containing toothpastes in the treatment of dentin hypersensitivity	30	Above 18	Thermal, tactile or sweet/sour stimulation followed by VAS scores ≥6 on at least two tooth	At home	Stillman’s method of brushing using a soft toothbrush.	VAS–evaporative, thermal, and tactile stimulation	7.5% CSPS toothpaste (Shy-NM)	Fluorinol toothpaste (Elgydium Sensitive Toothpaste)	Baseline, two, three, four weeks	DH decreased significantly in both groups. However, fluorinol toothpaste performed better in the third and fourth weeks to tactile stimulation.
**Bala et al., 2019** [31]	Comparison of Commercially available Desensitizing Toothpastes in the Management of Dentin Hypersensitivity-A Randomized Controlled Clinical Trial	20	18–70	Evaporative stimulation followed by Schiff scores ≥2 for at least two tooth	At home	Brushing 2× daily	VAS–evaporative and tactile stimulation/Schiff–evaporative stimulation	Bioactive glass toothpaste (Sensodyne Rapid Relief Toothpaste)	Arginine toothpaste (Colgate Sensitive Pro-Relief Toothpaste)	Baseline, four weeks	Toothpastes with arginine and bioactive glass are effective in reducing DH without any statistical significance between groups.
**Maximiano et al., 2019** [32]	Nd:YAG laser and calcium sodium phosphosilicate prophylaxis paste in the treatment of dentin hypersensitivity: a double-blind randomized clinical study	70	18–65	Evaporative stimulation followed by VAS scores ≥4 on at least one tooth	In office	Applied with a rubber cup at low speed for 60 s	VAS–evaporative, tactile stimulation	15% CSPS prophylaxis paste (NovaMin^®^)	Nd:YAG laser/Nupro^®^ paste with placebo Nd:YAG laser	Baseline, five min, one and four weeks	All treatments reduced DH pain equally.
**Patel et al., 2019** [33]	A randomised clinical trial on the efficacy of 5% fluoro calcium phosphosilicate-containing novel bioactive glass toothpaste	75	18–70	Evaporative stimulation followed by VAS scores ≥5	At home	Tooth-brushing with modified bass technique using soft-bristle toothbrush	VAS–evaporative stimulation	5% FCPS toothpaste (BioMin-F^®^)	8% arginine and calcium carbonate toothpaste (/Pro-Argin^®^)/Placebo toothpaste	Pre-baseline, baseline (15 days), and post-baseline (1 month)	The FCPS toothpaste was reported to be more effective than the other toothpastes in treating DH.

DH: dentin hypersensitivity, VAS: visual analogue scale, CSPS: calcium sodium phosphosilicate, FCPS: fluoro calcium phosphosilicate, HAP: hydroxyapatite.

**Table 3:** Assessment of studies with risk of bias.

	**Randomization Process**	**Deviations from Intended Interventions**	**Missing Outcome Data**	**Measurement of Outcome Data**	**Selection of the Reported Result**	**Overall Risk of Bias Judgment**
**Amaechi et al. (2021) [27]**	Low risk	Some concerns	Some concerns	Low risk	Low risk	High risk
**Arshad et al. (2021) [28]**	Low risk	Low risk	Some concerns	Low risk	Low risk	Some concerns
**Ashwini et al. (2018) [29]**	Low risk	Low risk	Low risk	Low risk	Low risk	Low risk
**Bhowmik et al. (2021) [30]**	Some concerns	Low risk	Low risk	Low risk	Low risk	Some concerns
**Bala et al. (2019) [31]**	Some concerns	Low risk	Low risk	Some concerns	Low risk	High risk
**Maximiano et al. (2019) [32]**	Low risk	Low risk	Low risk	Low risk	Low risk	Low risk
**Patel et al. (2019) [33]**	Some concerns	Low risk	High risk	Low risk	Low risk	High risk

## Data Availability

All the data used in this study are published in the literature.
